# Level Set Segmentation of Medical Images Based on Local Region Statistics and Maximum a Posteriori Probability

**DOI:** 10.1155/2013/570635

**Published:** 2013-11-05

**Authors:** Wenchao Cui, Yi Wang, Tao Lei, Yangyu Fan, Yan Feng

**Affiliations:** ^1^School of Electronics and Information, Northwestern Polytechnical University, Xi'an 710072, China; ^2^College of Science, China Three Gorges University, Yichang 443002, China; ^3^School of Electronic and Information Engineering, Lanzhou Jiaotong University, Lanzhou 730070, China

## Abstract

This paper presents a variational level set method for simultaneous segmentation and bias field estimation of medical images with intensity inhomogeneity. In our model, the statistics of image intensities belonging to each different tissue in local regions are characterized by Gaussian distributions with different means and variances. According to maximum a posteriori probability (MAP) and Bayes' rule, we first derive a local objective function for image intensities in a neighborhood around each pixel. Then this local objective function is integrated with respect to the neighborhood center over the entire image domain to give a global criterion. In level set framework, this global criterion defines an energy in terms of the level set functions that represent a partition of the image domain and a bias field that accounts for the intensity inhomogeneity of the image. Therefore, image segmentation and bias field estimation are simultaneously achieved via a level set evolution process. Experimental results for synthetic and real images show desirable performances of our method.

## 1. Introduction

Image segmentation is an important and necessary step in various image processing and computer vision applications. However, due to the imperfection of the image acquisition process, intensity inhomogeneity (or bias field) is often seen in many real-world images, especially in medical images [1]. For example, the intensity inhomogeneity in magnetic resonance (MR) images usually manifests itself as a smooth intensity variation across the image [2]. Thus the resultant intensities of the same tissue vary with the locations of the tissue within the image. This can cause serious misclassifications when intensity-based segmentation algorithms are used. Therefore, intensity inhomogeneity has been challenging difficulty in image segmentation.

The level set method, originally used as numerical technique for tracking interfaces and shapes [3], has been increasingly applied to image segmentation in the past decades [4, 5]. Compared with the classical image segmentation methods such as edge detection, thresholding, and region growing, level set methods have three desirable advantages. First, they can achieve subpixel accuracy of object boundaries [6]. Second, they allow incorporation of various prior knowledge, for example, shape and intensity distribution, so as to get more robust segmentation [7, 8]. Third, they can provide smooth and closed contours as segmentation results, which are necessary and can be readily used for further applications such as shape analysis and recognition [9]. In general, the existing level set methods can be categorized into two classes: edge-based models [6, 10–12] and region-based models [9, 13–17].

Edge-based models typically use image gradient as an image-based force to attract the contour toward object boundaries. These models have been successfully used for general images with strong object boundaries, but they are generally sensitive to the initial conditions and may suffer from boundary leakage problem for medical images which typically contain weak boundaries. These drawbacks greatly limit their utilities for medical images. Region-based models use a certain region descriptor to guide the motion of the active contour. Therefore they are less sensitive to initial contours and have better performance for images with weak object boundaries. A typical example is the piecewise constant (PC) model proposed in [13]. This model assumes that image intensities are statistically homogeneous in each region and thus always fails to segment images with intensity inhomogeneity. Intensity inhomogeneity can be dealt with by more complicated models than PC model. Tsai et al. [16] and Vese and Chan [17] independently proposed two similar region-based models, widely known as piecewise smooth (PS) models, for segmentation of more general images. The PS models cast image segmentation as a problem of finding an optimal approximation of the original image by a piecewise smooth function. Although the PS models do not assume homogeneity of image intensities and therefore are able to segment images with intensity inhomogeneity to some extent, they are computationally too expensive.

Recently, local intensity information has been incorporated into level set methods to effectively handle intensity inhomogeneity [1, 9, 18–23]. For example, Li et al. [9] defined a region-scalable fitting (RSF) energy in terms of a contour and two fitting functions that locally approximate the image intensities on the two sides of the contours. Based on the multiplicative model of images with intensity inhomogeneity and a derived local intensity clustering property, Li et al. [21] presented a variational level set framework for simultaneous segmentation and bias correction. Similarly, Chen et al. [19] adopted localized *K*-means-type clustering to define an energy which contains the bias field as a variable, and thus the energy minimization can also implement image segmentation and bias field estimation simultaneously. These models essentially draw upon local intensity means, which enable them to cope with intensity inhomogeneity. However, the local intensity means do not provide enough information for accurate segmentation, especially in the presence of strong noise and intensity inhomogeneity [1]. Therefore, more complete statistical characteristics of local intensities recently have to be taken into account. For instance, Rosenhahn et al. [18] used both local intensity means and variances to characterize the local intensity distribution in their proposed level set method. However, the local intensity means and variances are defined empirically in their models. Wang et al. [1, 20] proposed a local Gaussian distribution fitting (LGDF) energy with a level set function and local means and variances as variables, in which the local intensity means and variances are strictly derived from a variational principle, instead of being defined empirically. However, LGDF model cannot estimate the bias field so as to correct the intensity inhomogeneity in the original image. An improved LGDF model has been proposed by Chen et al. [22] to implement image segmentation and bias field correction simultaneously. Although these two LGDF models were derived based on maximum *a posteriori* probability (MAP) rule, they assumed the *a priori* probability of each partition among all partitions is equiprobable; that in other words, the MAP rule actually reduces to maximum likelihood (ML) rule. In [23], based on MAP rule, Shahvaran et al. proposed a variational level set combined with Markov random field model for simultaneous segmentation and bias filed correction of MR images, in which the *a priori* probability was approximated by the normalized pseudolikelihood [24].

In this paper, we propose a new level set method based on local region statistics and MAP rule for simultaneous image segmentation and bias field estimation. Based on the additive model of images with intensity inhomogeneity, we describe image intensities belonging to each different tissue in local regions by Gaussian distributions with different means and variances. By using maximum *a posteriori* probability (MAP) and Bayes' rule, we first define a local energy for image intensities in a neighborhood around each pixel. The local energy is then integrated over the entire image domain to form a global energy, which is converted into a level set formulation. Minimization of this global energy is achieved by an interleaved process of level set evolution and estimation of the bias field. As an important application, our method can be used for accurate segmentation and bias correction of medical images in the presence of severe intensity inhomogeneity and noise.

The remainder of this paper is organized as follows.  Section 2 reviews three different models of image with intensity inhomogeneity and some relevant level set methods.  Section 3 gives the energy formulation of our method and its minimization in level set framework is presented in Section 4. In Section 5, experimental results are presented and analyzed using a set of synthetic and real images, followed by some discussions in Section 6. This paper is summarized in Section 7.

## 2. Background

### 2.1. Models of Image with Intensity Inhomogeneity

According to [2, 25], three commonly used models of image with intensity inhomogeneity have been proposed in the literature, depending on how the true image *I*, bias field *b*, and noise *n* interact. Let *J* be the observed image; the first model assumes that the noise only arises from the scanner and is therefore independent of the bias field, which is defined as

(1) $$\begin{array}{c} J=I\cdot b+n. \end{array}$$

In the second model, only biological noise is considered, which is scaled by the bias field *b*, so that the signal to noise ratio (SNR) is preserved

(2) $$\begin{array}{c} J=\left(I+n\right)\cdot b. \end{array}$$

The third model is based on log-transformed intensities, by which the multiplicative bias field becomes additive. However, if two main sources of noise, namely, the biological noise and the scanner noise, should be considered in log-space, the noise formation will be nontrivial and unknown. Therefore, for methodological convenience, the noise is still assumed to be additive Gaussian [2]. The image model is expressed as

(3) $$\begin{array}{c} \log J=\log I+\log b+n. \end{array}$$

### 2.2. Li's Method

A generally accepted assumption on the bias field *b* is that it is smooth or slowly varying. Ideally, the intensity *I* belonging to the *i*th tissue should take a specific value *c*_*i*_, which represents the measured physical property [21]. Based on these two properties and the image model (1), Li et al. [21] firstly defined a local objective function to classify the data *J*(*x*) in the circular neighborhood *O*_*y*_ centered on *y* into *N* clusters using a *K*-means clustering method, and then this local objective function is integrated with respect to the neighborhood center over the entire image domain Ω to formulate the proposed energy function as follows.

(4) $$\begin{array}{c} E_{\text{Li}}=\int_{\Omega }\left(\overset{N}{\underset{i=1}{\sum }}\int_{\Omega_{i}}\omega \left(x-y\right){\left|J\left(x\right)-b\left(y\right)c_{i}\right|}^{2}dx\right)dy, \end{array}$$

where {*Ω*_*i*_}_*i*=1_^*N*^ denote a partition of the image domain Ω, *b*(*y*)*c*_*i*_ is the *i*th cluster center within the neighborhood *O*_*y*_, *ω* is a truncated Gaussian kernel which controls the size of the neighborhood *O*_*y*_. Note that the term inside the outer brackets is the local objective function in the neighborhood *O*_*y*_.

### 2.3. Chen's Method

Similar to Li's method, Chen et al. [19] applied the *K*-means clustering to classify the log-transformed intensities and thus proposed the following energy function:

(5) $$\begin{array}{c} E_{\text{Chen}}=\int_{\Omega }\left(\overset{N}{\underset{i=1}{\sum }}\int_{\Omega_{i}}\omega \left(x-y\right){\left|\tilde{J}\left(x\right)-\tilde{b}\left(y\right)-{\tilde{c}}_{i}\right|}^{2}dx\right)dy, \end{array}$$

where $\tilde{J}$, $\tilde{b}$ represent log⁡*J* and log⁡*b*, respectively. ${\tilde{c}}_{i}$ is the log-transformed value of the intensity *I* belonging to the *i*th tissue. Correspondingly, $\left(\tilde{b}(y)+{\tilde{c}}_{i}\right)$ is the *i*th cluster center within the neighborhood *O*_*y*_.

In essence, both Li's and Chen's methods utilize the local intensity means, and hence they can handle intensity inhomogeneity. However, the local intensity means do not provide enough information for accurate segmentation, especially in the presence of strong noise and intensity inhomogeneity. Besides, these two methods ignore the noise, which is also a key factor to influence the segmentation accuracy.

### 2.4. Chen's Improved Method

For the image model (2), taking the logarithmic transformation of both sides, we have

(6) $$\begin{array}{c} \log J=\log\left(I+n\right)+\log b. \end{array}$$

Let $\tilde{J}$, $\tilde{I}$, and $\tilde{b}$ denote log⁡*J*, log⁡(*I* + *n*), and log⁡*b*, respectively. Equation (6) can be written as

(7) $$\begin{array}{c} \tilde{J}=\tilde{I}+\tilde{b}. \end{array}$$

Assuming that the intensities $\tilde{I}$ of different tissue regions within the neighborhood *O*_*y*_ have different Gaussian distributions, that is, means and variances, Chen et al. [22] utilized MAP rule (strictly speaking, ML rule) to derive the local Gaussian distribution fitting energy as follows:

(8) $$\begin{array}{c} E_{\text{Chen}}^{*}=\int_{\Omega }\overset{N}{\underset{i=1}{\sum }}\int_{\Omega_{i}}-\;\omega \left(x-y\right)\log p_{i,y}\left(\tilde{J}\left(x\right)-\tilde{b}\left(y\right)\right)dx\;dy, \end{array}$$

where *p*_*i*,*y*_ $\left(\tilde{I}(x)\right)$ is the probability density of the *i*th region within the neighborhood *O*_*y*_, which is defined as

(9) $$\begin{array}{c} p_{i,y}\left(\tilde{I}\left(x\right)\right)=\frac{1}{\sqrt{2\pi }\sigma_{i,y}}\exp\left(-\frac{{\left(\tilde{I}\left(x\right)-u_{i}\left(y\right)\right)}^{2}}{2\sigma_{i,y}^{2}}\right), \end{array}$$

where *u*_*i*_(*y*), *σ*_*i*,*y*_ are intensity mean and standard deviation of the *i*th region within the neighborhood *O*_*y*_, respectively.

## 3. Our Proposed Energy Formulation

In this paper, we adopt the image model (3), which was used in [26–28]. Let $\tilde{J}$, $\tilde{I}$, and $\tilde{b}$ denote log⁡*J*, log⁡*I*, and log⁡*b*, respectively, (3) can be rewritten as

(10) $$\begin{array}{c} \tilde{J}=\tilde{I}+\tilde{b}+n, \end{array}$$

where noise *n* is assumed to be zero-mean Gaussian noise with variance *σ*_*i*_^2^ within the *i*th tissue domain.

To effectively exploit information on local intensities, we need to characterize the distribution of local intensities via partition of neighborhood as in [1]. For each point *y* in the image domain Ω, we consider a circular neighborhood with a small radius *r*, which is defined as *O*_*y*_ = {*x* : |*x* − *y*| ≤ *r*}. The partition {*Ω*_*i*_}_*i*=1_^*N*^ (*N* is the total number of segmented regions) of the entire domain Ω induces a partition of the neighborhood *O*_*y*_, that is, {*O*_*y*_∩*Ω*_*i*_}_*i*=1_^*N*^ forms a partition of *O*_*y*_ [1, 19]. For a slowly varying bias field $\tilde{b}$, the values $\tilde{b}(x)$ for all *x* in the circular neighborhood *O*_*y*_ can be well approximated by its value $\tilde{b}(y)$ at the center of *O*_*y*_, that is

(11) $$\begin{array}{c} \tilde{b}\left(x\right)\approx \tilde{b}\left(y\right),\;x\in O_{y}. \end{array}$$

If we assume that the intensity $\tilde{I}$ belonging to the *i*th tissue should take a specific value *c*_*i*_ as in [19], the intensities $\tilde{J}(x)$ within subregion *O*_*y*_∩*Ω*_*i*_ can be described as a Gaussian distribution with mean ($c_{i}+\tilde{b}(y)$) and variance *σ*_*i*_^2^, according to (10). In other words, the probability density in subregion *O*_*y*_∩*Ω*_*i*_ is

(12) $$\begin{array}{c} p_{i,y}\left(\tilde{J}\left(x\right)\right)=\frac{1}{\sqrt{2\pi }\sigma_{i}}\exp\left(-\frac{{\left(\tilde{J}\left(x\right)-\tilde{b}\left(y\right)-c_{i}\right)}^{2}}{2\sigma_{i}^{2}}\right). \end{array}$$

Now we consider the segmentation of the circular neighborhood *O*_*y*_ based on maximum *a posteriori* probability (MAP) rule. Let $p(x\in O_{y}\cap \Omega_{i}|\tilde{J}(x))$ be the *a posteriori* probability of the subregion *O*_*y*_∩*Ω*_*i*_ given the neighborhood gray values $\tilde{J}(x)$. According to Bayes' rule:

(13) p(x∈Oy∩Ωi ∣ J~(x))  =p(J~(x) ∣ x∈Oy∩Ωi)p(x∈Oy∩Ωi)p(J~(x)),

where $p(\tilde{J}(x)|x\in O_{y}\cap \Omega_{i})$ is the probability density in region *O*_*y*_∩*Ω*_*i*_, which is denoted by $p_{i,y}(\tilde{J}(x))$ in (12). *p*(*x* ∈ *O*_*y*_∩*Ω*_*i*_) is the *a priori* probability of the partition *O*_*y*_∩*Ω*_*i*_ among all partitions of *O*_*y*_, which can be denoted by *p*_*i*_(*y*). The *a priori* probability $p(\tilde{J}(x))$ is independent of the choice of the region. Therefore, (13) can be simplified as

(14) $$\begin{array}{c} p\left(x\in O_{y}\cap \Omega_{i}\;|\;\tilde{J}\left(x\right)\right)\propto p_{i,y}\left(\tilde{J}\left(x\right)\right)p_{i}\left(y\right). \end{array}$$

Assuming that the pixels within each region are independent, the MAP can be achieved by finding the maximum of $\prod_{i=1}^{N}\prod_{x\in O_{y}\cap \Omega_{i}}\left[p_{i,y}\left(\tilde{J}(x)\right)p_{i}\left(y\right)\right]$. Taking a logarithm, the maximization can be converted into the minimization of the following energy:

(15) $$\begin{array}{c} E_{y}=\overset{N}{\underset{i=1}{\sum }}\overset{}{\underset{O_{y}\cap \Omega_{\;i}}{\int }}-\log\left[p_{i,y}\left(\tilde{J}\left(x\right)\right)p_{i}\left(y\right)\right]dx. \end{array}$$

Generally, pixels far away from the neighborhood center *y* are expected to have less influence than pixels close to *y*. Therefore, we incorporate a nonnegative and monotonically decreasing weighting function *ω*(*d*) into the energy (15) to constrain the influence of different pixels. As in [1, 9, 19–23], we choose a truncated Gaussian kernel as the weighting function *ω*; that is,

(16) $$\begin{array}{c} \omega \left(d\right)=\{\begin{array}{cc} \frac{1}{a}e^{-{\left|d\right|}^{2}/2\sigma^{2}} & \left|d\right|\leq r,\\ 0 & \text{else}, \end{array} \end{array}$$

where *σ* is the standard deviation of the Gaussian kernel and *a* is a constant to normalize the Gaussian kernel. With this weighting function, the above objective function *E*_*y*_ can be rewritten as

(17) $$\begin{array}{c} E_{y}=\overset{N}{\underset{i=1}{\sum }}\int_{\Omega_{i}}-\omega \left(x-y\right)\log\left[p_{i,y}\left(\tilde{J}\left(x\right)\right)p_{i}\left(y\right)\right]dx \end{array}$$

as *ω*(*x* − *y*) = 0 for *x* ∉ *O*_*y*_.

The ultimate goal is to minimize *E*_*y*_ for all the center points *y* in the image domain *Ω*, which directs us to define the following double integral energy:

(18) E=∫ΩEydy=∫Ω(∑i=1N∫Ω  i−ω(x−y)            ×log⁡[pi,y(J~(x))pi(y)]dx)dy.

Substituting (12) into (18), we can obtain the proposed energy formulation as follows:

(19) E=∫Ω(∑i=1N∫Ωiω(x−y)       ×(−log⁡(pi(y))+log⁡(2πσi)       +(J~(x)−b~(y)−ci)22σi2)dx)dy.

Note that our method is different from the method proposed in [23]. First, the image model of intensity inhomogeneity we use is based on log-transformed intensities. Second, we incorporate the *a priori* probability as a variable into the energy function; thus the optimal value will be found by the variational principle. 

## 4. Energy Minimization in Level Set Framework

The proposed energy *E* in (19) is expressed in terms of the regions *Ω*_1_,…, *Ω*_*N*_. It is difficult to derive a solution to the energy minimization problem from this expression of *E*. Alternatively, we can use one or multiple level set functions to represent the disjoint regions *Ω*_1_,…, *Ω*_*N*_. Thus this energy *E* can be converted into an equivalent level set formulation, which can be solved by using well-established variational methods [29]. Besides, the energy *E* is subject to the constraint ∑_*i*=1_^*N*^ *p*_*i*_(*y*) = 1; therefore, its minimization can be derived using the Lagrange multiplier method.

### 4.1. Two-Phase Level Set Formulation

We assume that the image domain can be partitioned into two regions corresponding to the object and background; that is, *N* = 2. These two regions can be represented as the regions outside and inside the zero level set of function *ϕ*; that is, *Ω*_1_ = {*ϕ* > 0} and *Ω*_2_ = {*ϕ* < 0}. Using the Heaviside function *H*, the energy *E* in (19) can be expressed as

(20) E(ϕ,c1,c2,σ1,σ2,b~,p1,p2)=∫Ω(∑i=12∫Ωω(x−y)     ×(−log⁡(pi(y))+log⁡(2πσi)         +(J~(x)−b~(y)−ci)22σi2)Mi(ϕ(x))dx)dy,

where *M*_1_(*ϕ*(*x*)) = *H*(*ϕ*(*x*)) and *M*_2_(*ϕ*(*x*)) = 1 − *H*(*ϕ*(*x*)). Heaviside function *H* is usually approximated by a smoothing function *H*_*ε*_ defined by

(21) $$\begin{array}{c} H_{\varepsilon }\left(x\right)=\frac{1}{2}\left[1+\frac{2}{\pi }\arctan\left(\frac{x}{\varepsilon }\right)\right], \end{array}$$

with *ε* = 1 as in [13]. The derivative of *H*_*ε*_ is the following smoothing function:

(22) $$\begin{array}{c} \delta_{\varepsilon }\left(x\right)=H_{\varepsilon }^{\prime }\left(x\right)=\frac{1}{\pi }\frac{\varepsilon }{\varepsilon^{2}+x^{2}}. \end{array}$$

As in typical level set methods, we need to regularize the zero level set by penalizing its length to obtain a smooth contour during evolution. This can be realized by the length term

(23) $$\begin{array}{c} L\left(\phi \right)=\int \left|\nabla H\left(\phi \left(x\right)\right)\right|dx. \end{array}$$

To avoid the expensive reinitialization procedures, we regularize the level set function by penalizing its deviation from a signed distance function as in [30], characterized by the following energy:

(24) $$\begin{array}{c} R\left(\phi \right)=\int \frac{1}{2}{\left(\left|\nabla \phi \left(x\right)\right|-1\right)}^{2}dx. \end{array}$$

Therefore, the entire energy functional is defined as

(25) F(ϕ,c1,c2,σ1,σ2,b~,p1,p2)  =E(ϕ,c1,c2,σ1,σ2,b~,p1,p2)+νL(ϕ)+μR(ϕ),

where *ν*, *μ* are positive constants.

The minimization of energy *F* can be achieved by an iterative process: in each iteration, we minimize the energy *F* with respect to each of its variables *ϕ*, *c*_*i*_, *σ*_*i*_, $\tilde{b}$, and *p*_*i*_, given the other four updated in previous iteration. We give the solution to the energy minimization with respect to each variable as follows.

For fixed *c*_*i*_, *σ*_*i*_, $\tilde{b}$, and *p*_*i*_, the minimization of *F* in (25) with respect to *ϕ* can be achieved by using standard gradient descent method, namely, solving the gradient flow equation

(26) $$\begin{array}{c} \frac{\partial \phi }{\partial t}=-\frac{\partial F}{\partial \phi }, \end{array}$$

where ∂*F*/∂*ϕ* is the Gâteaux derivative of the energy *F*.

By calculus of variations, we can compute the Gâteaux derivative ∂*F*/∂*ϕ* and express the corresponding gradient flow equation as

(27) ∂ϕ∂t=−δε(ϕ)(e1−e2)+vδε(ϕ)div⁡(∇ϕ|∇ϕ|) +μ(∇2ϕ−div⁡(∇ϕ|∇ϕ|)),

where ∇ is the gradient operator, div⁡(·) is the divergence operator, ∇^2^ is the Laplace operator, and

(28) ei(x)=∫Ωω(x−y)  ×[−log⁡(pi(y))+log⁡(2πσi)    +(J~(x)−b~(y)−ci)22σi2]dy.

For fixed *ϕ*, *σ*_*i*_, $\tilde{b}$, and *p*_*i*_, the optimal *c*_*i*_ that minimizes the energy *F* is given by

(29) $$\begin{array}{c} c_{i}=\frac{\iint_{\Omega }^{}\omega \left(x-y\right)\left(\tilde{J}\left(x\right)-\tilde{b}\left(y\right)\right)M_{i}\left(\phi \left(x\right)\right)dx\;dy}{\iint_{\Omega }^{}\omega \left(x-y\right)M_{i}\left(\phi \left(x\right)\right)dx\;dy}.\;\; \end{array}$$

Similarly, we can obtain

(30) σi=∬Ωω(x−y)(J~(x)−b~(y)−ci)2Mi(ϕ(x))dx dy∬Ωω(x−y)Mi(ϕ(x))dx dy,

(31) b~(y)=∫Ω∑i=1Nω(x−y)((J~(x)−ci)/σi2)Mi(ϕ(x))dx∫Ω∑i=1Nω(x−y)(1/σi2)Mi(ϕ(x))dx,

(32) pi(y)=∫Ωω(x−y)Mi(ϕ(x))dx∑i=1N∫Ωω(x−y)Mi(ϕ(x))dx.

### 4.2. Multiphase Level Set Formulation

For the case of *N* ≥ 3, we can use multiple level set functions *ϕ*_1_,…, *ϕ*_*n*_ to represent multiple regions {*Ω*_*i*_}_*i*=1_^*N*^ with *N* ≤ 2^*n*^ as in [17]. For notational simplicity, we denote Φ = (*ϕ*_1_,…, *ϕ*_*n*_), *C* = (*c*_1_,…, *c*_*N*_), Σ = (*σ*_1_,…, *σ*_*N*_), and *P* = (*p*_1_,…, *p*_*N*_). The energy for general multiphase formulation of our method can be defined as

(33) F(Φ,C,Σ,b~,P) =∫Ω(∑i=1N∫Ωω(x−y)(−log⁡(pi(y))+log⁡(2πσi)                                  +(J~(x)−b~(y)−ci)22σi2)         ×Mi(Φ(x))dx)dy  +ν∑j=1nL(ϕj)+μ∑j=1nR(ϕj),

where *M*_*i*_(Φ) are membership functions of the regions *Ω*_*i*_, *i* = 1,…, *N*, such that

(34) $$\begin{array}{c} M_{i}\left(\phi_{1}\left(x\right),\ldots ,\phi_{n}\left(x\right)\right)=\{\begin{array}{cc} 1 & x\in \Omega_{i}\\ 0 & \text{else}. \end{array} \end{array}$$

For example, in the case of *N* = 3, we use two level set functions *ϕ*_1_ and *ϕ*_2_ to define *M*_1_(*ϕ*_1_, *ϕ*_2_) = *H*(*ϕ*_1_)*H*(*ϕ*_2_), *M*_2_(*ϕ*_1_, *ϕ*_2_) = *H*(*ϕ*_1_)(1 − *H*(*ϕ*_2_)), and *M*_3_(*ϕ*_1_, *ϕ*_2_) = 1 − *H*(*ϕ*_1_) to give a three-phase level set formulation of our method. For *N* = 4, *M*_*i*_(Φ) can be defined as *M*_1_(*ϕ*_1_, *ϕ*_2_) = *H*(*ϕ*_1_)*H*(*ϕ*_2_), *M*_2_(*ϕ*_1_, *ϕ*_2_) = *H*(*ϕ*_1_)(1 − *H*(*ϕ*_2_)), *M*_3_(*ϕ*_1_, *ϕ*_2_) = (1 − *H*(*ϕ*_1_))*H*(*ϕ*_2_), and *M*_4_(*ϕ*_1_, *ϕ*_2_) = (1 − *H*(*ϕ*_1_))(1 − *H*(*ϕ*_2_)).

The minimization of the energy *F* in (33) with respect to the variable Φ = (*ϕ*_1_,…, *ϕ*_*n*_) can be performed by solving the following gradient flow equations

(35) ∂ϕ1∂t=−∑i=1N∂Mi(Φ)∂ϕ1ei+νδε(ϕ1)div⁡(∇ϕ1|∇ϕ1|)   +μ(∇2ϕ1−div⁡(∇ϕ1|∇ϕ1|))⋮∂ϕn∂t=−∑i=1N∂Mi(Φ)∂ϕnei+νδε(ϕn)div⁡(∇ϕn|∇ϕn|)   +μ(∇2ϕn−div⁡(∇ϕn|∇ϕn|)).

The energy minimization with respect to *c*_*i*_, *σ*_*i*_, $\tilde{b}$, and *p*_*i*_ can be achieved by the same procedure as in the two-phase case. And it is easy to show that optimal *c*_*i*_, *σ*_*i*_, $\tilde{b}$, and *p*_*i*_ are given by (29), (30), (31), and (32), respectively, only by replacing *M*_*i*_(*ϕ*) with *M*_*i*_(Φ).

The implementation procedure of our proposed method can be summarized as follows:

**Step 1**

Initialize the level set function *ϕ* (or Φ), the bias field $\tilde{b}$, and the *a priori* probability *p*_*i*_.

**Step 2**

Update *c*_*i*_ and *σ*_*i*_ using (29) and (30), respectively.

**Step 3**

Update the level set functions *ϕ* (or Φ) according to (27) (or (35)).

**Step 4**

Update the bias field $\tilde{b}$ using (31).

**Step 5**

Update the *a priori* probability *p*_*i*_ using (32).

**Step 6**

Repeat Steps 2–5 until the convergence criteria are met.

## 5. Experimental Results

In this section, the proposed method has been tested on both synthetic and real images from different modalities. The level set function *ϕ* can be simply initialized as a binary step function as in [9], which takes a negative constant value −*c*_0_ (e.g., *c*_0_ = 2) inside a region *R*_0_ and a positive constant value +*c*_0_ outside it. The bias field $\tilde{b}(y)$ is initially equal to zero for all *y*. The a priori probability *p*_*i*_ is initially equiprobable. For the experiments in this paper, we set the iteration time step Δ*t* = 0.1, standard deviation of the Gaussian kernel *σ* = 4, neighborhood radius of the Gaussian kernel *r* = 15, weighting coefficients *μ* = 1.0 and *v* = 0.001 × 255^2^ for all images on a trial basis. 

### 5.1.  Application to Synthetic Images

**Figure 1:**
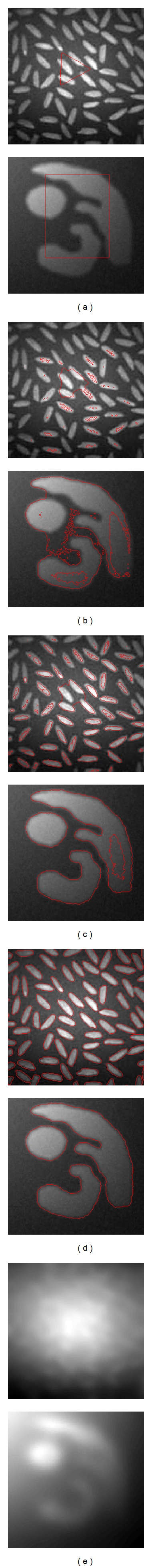
Application of our method to synthetic images. Column 1: original images and initial contours. Columns 2-3: intermediate contours. Column 4: final contours. Column 5: the estimated bias fields.

We first apply our method to segment two synthetic images, which are displayed in the first column of Figure 1. These two images are corrupted by strong noise and intensity inhomogeneity. The initial contours superposed on the original images are chosen manually. The intermediate contours obtained by running our method are shown in the second and third columns, and the final contours obtained after the convergence of our algorithm are shown in the fourth column. The two images in the fifth column are the estimated bias fields. It is revealed from Figure 1 that the new contours gradually emerge during the evolution process. In the final segmentation results, the complete boundaries of multiple objects can be successfully extracted despite of the impact of noise and intensity inhomogeneity.

### 5.2.  Application to Real Medical Images

**Figure 2:**
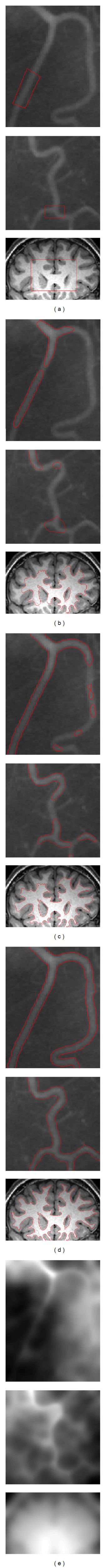
Application of our method to real medical images. Column 1: original images and initial contours. Columns 2-3: intermediate contours. Column 4: final contours. Column 5: the estimated bias fields.

We also evaluate the performance of the proposed method on a set of real medical images. The first two rows of Figure 2 show two X-ray vessel images with intensity inhomogeneity. In addition, parts of the vessel boundaries are quite weak. Such properties render it a nontrivial task to segment the vessels in the images. A coronal slice of brain MR image is shown in the third row of Figure 2, in which intensity inhomogeneity is quite obvious. The original images and initial contours, the intermediate results, the final results, and the estimated bias fields are shown from the left column to the right column of Figure 2. These satisfactory segmentation results demonstrate desirable performance of our method for these challenging medical images.

### 5.3.  MR Image Segmentation and Bias Correction

**Figure 3:**
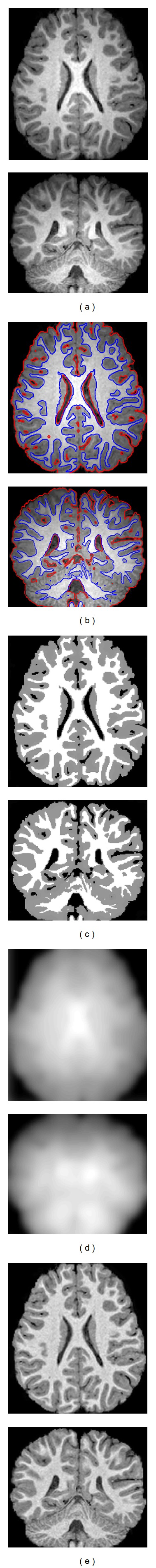
Application of our method to 3T brain MR images. Column 1: original images. Column 2: final contours. Column 3: segmentation results. Column 4: the estimated bias fields. Column 5: the bias corrected images. The red and blue curves are zero level sets of *ϕ*_1_ and *ϕ*_2_, respectively.

**Figure 4:**
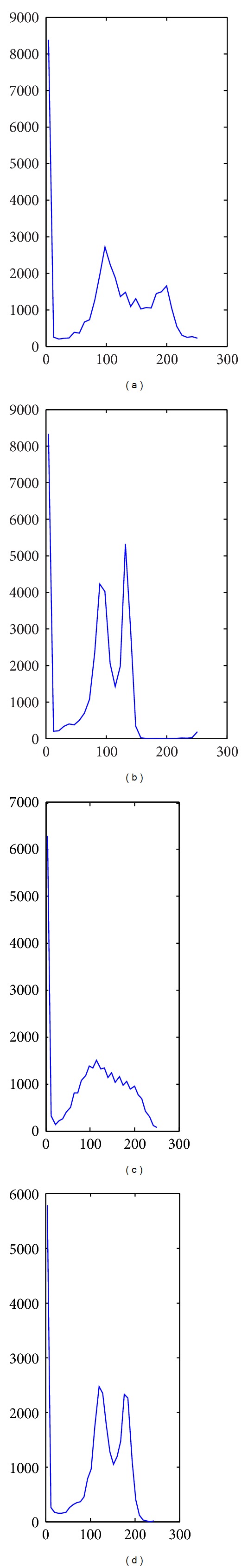
Histograms of original images and bias corrected images. (a) and (c) Histograms of the original images corresponding to the first column in Figure 3. (b) and (d) Histograms of the bias corrected images corresponding to the fifth column in Figure 3.

We then focus on the application of the proposed method, which is implemented using three-phase model, to segmentation and bias correction of brain MR images. The first column of Figure 3 shows two 3T MR images with obvious intensity inhomogeneity. Some parts of white matter (WM) and gray matter (GM) boundaries are quite fuzzy due to low contrast. In addition, the noise also causes certain difficulties. However, from the final contours and the segmentation results, respectively, shown in the second and third columns of Figure 3, it is obvious that our method successfully extracts the WM and GM in spite of the problems. The estimated bias fields and the bias corrected images are shown in the fourth and fifth columns. It can be seen that the intensities within each tissue become quite homogeneous in the bias corrected images. The improvement of the image quality in term of intensity homogeneity can be also demonstrated by comparing the histograms of the original images and the bias corrected images in Figure 4. Obviously, there are three well-defined and well-separated peaks in the histograms of the bias corrected images, each corresponding to a tissue or the background in the images. In contrast, the histograms of the original images do not have such well-separated peaks due to the mixture of the intensity distribution caused by the bias field.

### 5.4.  Comparison with Other Methods

In this subsection, we first compare the proposed method with Chen's method, Li's method, and Chen's improved method.

**Figure 5:**
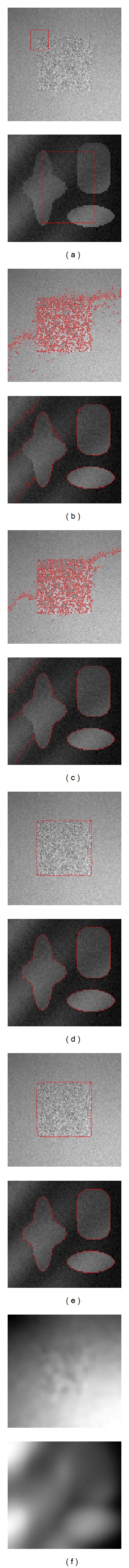
Comparison with other methods using synthetic images. Column 1: original images and initial contours. Column 2: Chen's method. Column 3: Li's method. Column 4: Chen's improved method. Column 5: our method. Column 6: the estimated bias fields obtained by our method.

Figure 5 shows the results of four algorithms for two synthetic images with intensity inhomogeneity. The first image contains a square object and background of the same intensity means but different variances. Since only the local intensity means are taken into account, Chen's and Li's methods fail to locate the object boundaries. By contrast, Chen's improved method and our method, which utilize local intensity means and variances efficiently, can differentiate the object and background using local intensity variance information. The segmentation task for the second image is to extract three objects with different shape. Due to the complicated intensity inhomogeneity and strong noise, Chen's and Li's models fall into a local minimum and thus obtain unsatisfactory segmentation results. Nevertheless, both Chen's improved method and our method successfully implement the segmentation task.

**Figure 6:**
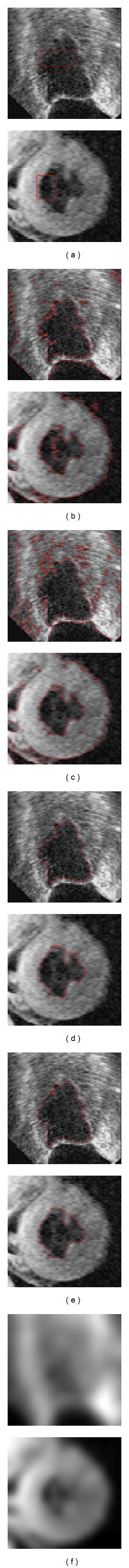
Comparison with other methods using ultrasound images. Column 1: original images and initial contours. Column 2: Chen's method. Column 3: Li's method. Column 4: Chen's improved method. Column 5: our method. Column 6: the estimated bias fields obtained by our method.

Figure 6 presents the results for two ultrasound images. The original images have severe intensity inhomogeneity and strong noise. Chen's and Li's methods are unable to overcome these handicaps, and hence the segmentation results have many false contours. Because both Chen's improved method and our method fully exploit intensity distribution information in local regions, their segmentation results successfully separate the lesion from normal tissues.

**Figure 7:**
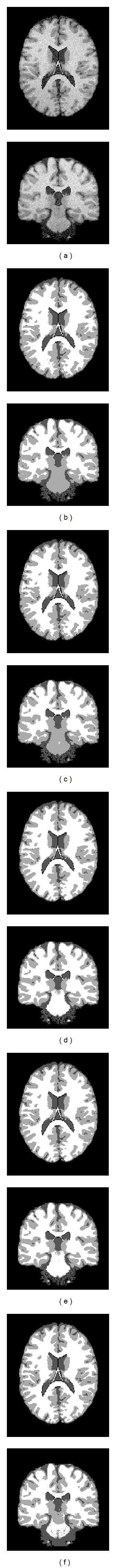
Comparison of segmentation results on two simulated brain MR images. Column 1: original images. Column 2: Chen's method. Column 3: Li's method. Column 4: Chen's improved method. Column 5: our method. Column 6: ground truth.

 Next, in order to quantitatively compare the proposed method with the above-mentioned other three methods, we use 20 T1-weighted simulated brain MR images with ground truth from Brain-Web in the link http://www.bic.mni.mcgill.ca/brainweb/. All the MR images are selected from the same volume data with 40% image intensity inhomogeneity and 7% noise. The task of segmentation is to partition the brain MR images into four regions, that is, white matter (WM), gray matter (GM), cerebral spinal fluid (CSF), and background. The segmentation results obtained by applying these methods to two sample images are shown in Figure 7. It can be observed that in contrast with Chen's improved method and our method, both Chen's and Li's methods misclassify more WM into GM, especially in the lower part of the image in the second row of Figure 7. We adopt the Jaccard similarity (*JS*) [2] as a measurement of the segmentation accuracy. The *JS* between two regions *S*_1_ and *S*_2_ is defined as *JS*(*S*_1_, *S*_2_) = |*S*_1_∩*S*_2_ | /|*S*_1_ ∪ *S*_2_|. We compute the *JS* between the segmented region *S*_1_ obtained by the algorithm and the corresponding region *S*_2_ given by the ground truth. The closer the *JS* value to 1, the better the segmentation result. The resulting average *JS* values over 20 images for these four methods are listed in Table 1. It demonstrates that both Chen's improved method and our method, which use local means and variances, achieve higher accuracy than the other two methods which only utilize local means. However, Chen's improved method virtually derived from ML rule ignores the *a priori* information with respect to each partition and therefore has lower accuracy than our method which is really based on MAP rule. 

**Figure 8:**
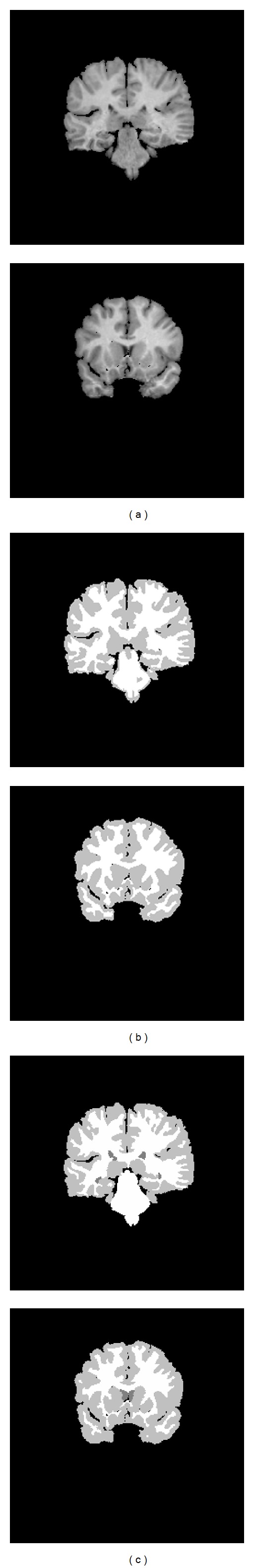
Application of our method to two real IBSR images. Column 1: original images. Column 2: segmentation results. Column 3: ground truth.

 Finally, we also quantitatively evaluate the performance of our method for 20 real MR images selected from the Internet Brain Segmentation Repository (IBSR). The data sets are available at http://www.nitrc.org/projects/ibsr. The test images are selected from sequence 100_23. The expert manual segmentation results provide the basis for a “ground truth” set to be used for comparative study.  Figure 8 shows the segmentation results of our method in two sample images and corresponding ground truth. We compare the accuracy of our method with that of six segmentation methods provided by the IBSR, including the MAP, adaptive MAP, bias MAP, FCM, maximum likelihood, and tree-structure *K*-means algorithm. Furthermore, two additional methods, FSL-FAST [31] and SPM [25], which report average results on IBSR data set, are also included. The average *JS* values of nine methods are shown in Table 2. It can be seen that our method outperforms other eight methods in terms of segmentation accuracy.

## 6. Discussion

Our proposed method is based on log-transformed data, and hence low intensity pixels have to be excluded to avoid numerical problems [25]. As in other level set methods, there are parameters which need to be adjusted to obtain appropriate segmentation results. The parameter *μ* in (25) or (33) influences the regularization term of the level set, and its effect has been detailed in [30]. As shown in [22], the parameter *ν* can be adjusted to smooth the curves, in a way that the smoothness of the curve increases when *ν* increases. The truncated Gaussian kernel *ω*(*x* − *y*) is usually constructed as a *m* × *m* mask (or *m* × *m* × *m* for 3D data), where *m* is a small odd number typically within 3*σ* ~ 5*σ*. In our experiments, we find that *σ* = 4 can appropriately complete segmentation task for all the images.

In addition, it can be observed from (28)–(32) that for every iteration, a few convolution operations are performed. The convolution operations require a high computational complexity *O*(*S*_1_ *S*_2_), where *S*_1_ and *S*_2_ are the size of the image and the Gaussian kernel, respectively. For the images with the size 181 × 217, the average CPU time using Matlab codes on a PC with Intel Dual-Core 2.93 GHz Processor and 2G RAM is about 20 s. For the 3D data with the size 256 × 256 × 20 from the IBSR, the average CPU time is about 20 minutes. Obviously, our method must be computationally expensive to segment the whole brain. However, with the initial curve near the edge of the objects, the proposed method may need less iterations and less CPU time.

## 7. Conclusion

In this paper, we have proposed a new level set method for segmentation and bias field estimation of images with intensity inhomogeneity. Based on local region statistics and maximum a posteriori probability, we define an energy in terms of the level set functions that represent a partition of the image domain and a bias field that accounts for the intensity inhomogeneity of the image. Our method can estimate intensity inhomogeneity, handle noise efficiently, and also allow flexible initialization. In addition, the regularity of the level set function is intrinsically preserved by the level set regularization term to ensure an accurate computation avoiding expensive reinitialization procedures. Comparisons with other approaches demonstrate the superior performance of the proposed method. However, our proposed method does have some limitations such as high computation cost. In our future work, we plan to reduce the computational scheme by using the Split Bregman method [32], instead of gradient descent method used in this paper.

## Figures and Tables

**Table 1:** Comparison of the segmentation results obtained by the four methods over 20 images from the BrainWeb (mean ± SD).

Tissue	Chen's method	Li's method	Chen's improved method	Proposed method
WM	0.8613 ± 0.0254	0.8792 ± 0.0223	0.9011 ± 0.0147	0.9289 ± 0.0152
GM	0.7583 ± 0.0317	0.7723 ± 0.0298	0.7953 ± 0.0241	0.8128 ± 0.0248
CSF	0.7349 ± 0.0414	0.7543 ± 0.0405	0.8311 ± 0.0319	0.8532 ± 0.0296

**Table 2:** The average *JS* values of the segmentation results obtained by the nine methods over the IBSR data set.

Segmentation Method	WM	GM
Maximum a posteriori probability (MAP)	0.554	0.550
Adaptive MAP	0.567	0.564
Bias MAP	0.562	0.558
Fuzzy c-means (FCM)	0.567	0.473
Maximum likelihood (ML)	0.551	0.535
Tree-structure *K*-means	0.571	0.477
FSL-FAST	0.541	0.510
SPM	0.670	0.652
Our method	0.801	0.823
